# Viperin: A Multifunctional Protein in Antiviral Immunity and Disease Pathogenesis

**DOI:** 10.3390/pathogens14050510

**Published:** 2025-05-21

**Authors:** Qun Cui, Ying Miao, Min Li, Hui Zheng, Yukang Yuan

**Affiliations:** 1 International Institute of Infection and Immunity, Institutes of Biology and Medical Sciences, Soochow University, Suzhou 215123, Chinamli3678@suda.edu.cn (M.L.); 2 Jiangsu Key Laboratory of Infection and Immunity, Soochow University, Suzhou 215123, China; 3 Department of Laboratory Medicine, Institute of Laboratory Medicine, Sichuan Provincial People’s Hospital, School of Medicine, University of Electronic Science and Technology of China, Chengdu 611731, China

**Keywords:** Viperin, interferon-stimulated gene, antiviral response, innate immunity, disease pathogenesis

## Abstract

Innate immunity is an important component of the immune system and serves as the first line of defense for the host against the invasion of foreign pathogens. Viperin (RSAD2), a core member of the interferon-stimulated gene (ISG) family, plays a key role in innate immunity through direct inhibition of viral replication and modulation of the host immune–metabolic network. The intracellular expression of Viperin rises markedly after viral infection or interferon-induced induction, showing a wide range of antiviral activities. In recent years, the versatility of Viperin in viral infections, autoimmune diseases, and tumor immune metabolism has been gradually revealed. Here, we summarize and discuss the gene regulatory network, molecular functions, and multi-dimensional roles of Viperin in diseases to provide a theoretical basis for the development of broad-spectrum antiviral strategies and immunometabolic therapies based on Viperin.

## 1. Introduction

Viral infections are one of the major threats to global public health, with pathogens such as influenza virus, HIV, HCV, and novel coronavirus (SARS-CoV-2) causing millions of infections and deaths each year [1]. The innate immune system is the host’s first line of defense against viral invasion, and its core mechanism relies on the interferon (IFN) system. IFN, as a functional protein in the host, plays an important antiviral role in intrinsic and adaptive immunity. However, it is not the IFN itself that exerts antiviral function, but rather the downstream effectors (interferon-stimulated genes (ISGs)) that are produced by IFN recognition and binding to cognate receptors, thereby activating cellular signaling [2,3,4,5]. IFN induces the expression of hundreds of ISGs by activating the JAK-STAT signaling pathway, resulting in a broad spectrum of antiviral effects [3]. ISGs play a key role by directly inhibiting viral replication or modulating host immune signaling pathways, for example, by inhibiting viral RNA polymerase activity or interfering with viral particle assembly [5]. However, viruses escape the surveillance of the interferon system through evolutionary mechanisms, such as blocking IFN signaling or degrading ISG products, creating a dynamic balance between host and pathogen [6]. This mechanism is particularly prominent in emerging infectious diseases such as COVID-19, where functional deficits in the IFN system correlate strongly with disease severity [7,8,9]. In recent years, high-throughput screening techniques have disclosed the functional diversity of ISGs. For example, Schoggins et al. identified through systematic screening that hundreds of ISGs possess specificity for targeting different viruses. Among them, Viperin (RSAD2) has drawn considerable attention because of its unique enzymatic activity and metabolic regulatory functions. It inhibits the replication of a broad spectrum of viruses, including dsDNA viruses, positive and negative single-stranded RNA viruses, and retroviruses in humans and other species. Examples include ZIKV, West Nile virus (WNV), Influenza A Virus (IAV), Human Immunodeficiency Virus 1 (HIV-1), and human cytomegalovirus (HCMV) [10,11,12,13,14]. Its encoded protein catalyzes the synthesis of ddhCTP through NADPH-dependent reductase activity and directly inhibits RNA virus genome replication [15]. This versatility makes it a key molecule in linking innate immunity to metabolic regulation.

Although important advances have been made in the antiviral mechanism of Viperin, its specific effects against different viruses, manifestations in diseases, and clinical translational potential still need to be systematically summarized. This review aims to integrate recent studies and analyze the gene regulation, molecular function, and role of Viperin in diseases to provide a theoretical basis for the development of Viperin-based antiviral strategies. For example, the broad-spectrum antiviral potential of ddhCTP, a product of Viperin’s enzymatic activity, its novel function in tumor immunity, and the association of its overactivation with autoimmune diseases, such as systemic lupus erythematosus [15,16], suggest that it holds great promise as a therapeutic target. By combing the multidimensional regulatory mechanisms of Viperin, this review will provide new ideas for antiviral drug design and immunotherapy optimization.

## 2. Structure and Expression Regulation of Viperin

**Figure 1:**
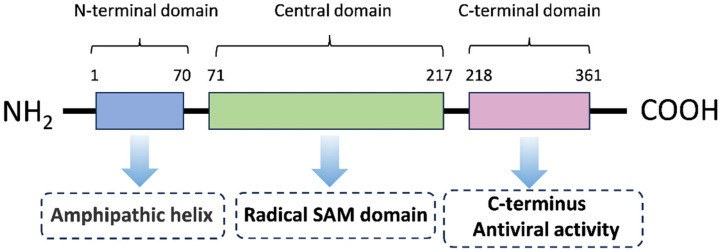
The primary structure of Viperin. The Viperin protein is composed of 361 amino acids and has three distinct regions in its primary structure: an N-terminus (which varies in length and sequence with species), a central conserved region, and a highly conserved C-terminus. The tertiary structure of Viperin is a bilayer structure containing both α-helices and β-sheets. The C-terminal region of Viperin is also highly conserved and has been demonstrated to be critical for its ability to limit the replication of a number of viruses.

Viperin (RSAD2) is a broad-spectrum antiviral protein induced by interferon [10,17] and is a member of the Radical S-adenosylmethionine (Radical SAM) superfamily, which are conserved proteins with a predicted molecular weight of 42 kDa [18]. It is approximately 361 amino acids in length and contains three major structural domains: a variable amino-terminal domain with an amphiphilic α-helix and a leucine zip domain, a conserved central domain containing a free-radical S-adenosylmethionine (SAM) domain, and a highly conserved C-terminal domain [18,19] (Figure 1). The N-terminal transmembrane domain consists of an amphipathic α-helix, which is responsible for anchoring Viperin to the cytoplasmic face of the endoplasmic reticulum (ER) and lipid droplets (LDs), a position that is essential for its antiviral function [20,21]. It has been found that the N-terminal domain of Viperin is crucial for the association between the endoplasmic reticulum and lipid droplets [20,22], and the amphiphilic helix at the N-terminus of Viperin is required for its antiviral activity against the release of chikungunya virus (CHIKV) and Equine Infectious Anemia Virus (EIAV) [21,23]. The SAM domain is central to the catalytic activity of Viperin and is involved in the regulation of lipid metabolism and viral suppression functions [24,25] and contains a [4Fe-4S] cluster responsible for the production of catalytically essential 5′-deoxyadenosine radicals from SAM. Mutations in the binding motif of the [4Fe-4S] cluster reduced the antiviral activity of Viperin against West Nile virus (WNV), Dengue virus (DENV), tick-borne encephalitis virus (TBEV), hepatitis C virus (HCV), Human Immunodeficiency Virus (HIV), and Bunyamwera virus (BUNV), suggesting that free radical SAM activity is critical for limiting viral replication [11,26,27,28,29,30]. In addition, the active center of Viperin contains several positively charged conserved residues, such as Arg193 and Arg211, which promote its catalytic activity by interacting with the phosphate group of the substrate CTP [31]. In recent years, it has been found that the C-terminal structural domain of Viperin also activates TRAF6-dependent ubiquitination signaling and enhances the type I interferon response [32]. The C-terminus of Viperin drives the accumulation of molecules that specifically antagonize viral polymerases and force strand termination during RNA viral replication [33]. These findings reveal the complexity and functional diversity of the Viperin gene structure [24,34].

**Figure 2:**
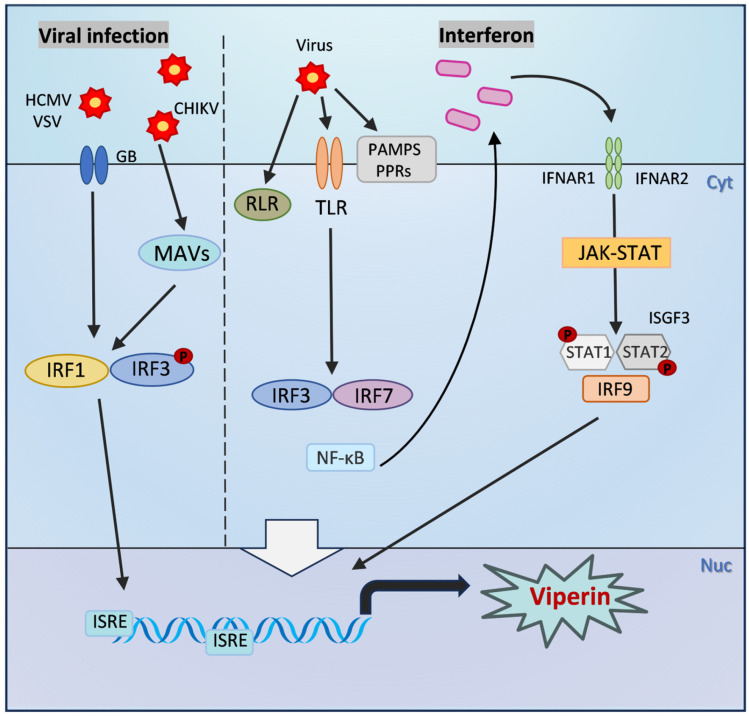
The inducible expression pathway of Viperin. After viral infection: PAMPs, PPRs, and RLR are activated, which in turn activates the phosphorylation of transcription factors NF-κB and interferon regulatory factors IRF3 and IRF7, and induces the production of type I interferons. Interferons can bind to type I interferon receptors on the cell surface in a paracrine and autocrine manner. The dimerization of IFNAR subunits activates the JAK-STAT signaling pathway, ultimately inducing the formation of ISGF3. ISGF3 can directly bind to the ISRE and promote the transcription of Viperin. Viperin is also produced independently of the interferon pathway and is directly regulated by IRF1 or IRF3. The membrane glycoprotein glycoprotein B (GB) of HCMV can induce the phosphorylation of IRF3 and bind to the ISRE upstream of the Viperin promoter, thereby directly initiating its transcription. VSV infection stimulates IRF1 to bind to the two proximal ISREs of the Viperin promoter, activating its transcription. CHIKV infection also directly induces Viperin expression by activating MAVS and downstream IRF3.

The expression of Viperin is mainly regulated by type I (IFN-α/β) and type II (IFN-γ) interferons through the classical JAK/STAT signaling pathway. Through the binding of interferon receptors to the cell surface, JAK kinases are activated and contribute to the formation of STAT1/STAT2 heterodimers, which further bind to IFN regulatory factors (IRFs) to initiate the expression of interferon-stimulated genes (ISGs) such as Viperin [3,35]. It is known that the interferon-stimulated response element (ISRE) is an enhancer that regulates gene expression, and the promoter of Viperin contains two ISRE binding sites, and ISGF3 can directly bind to the ISRE to promote Viperin transcription [36] (Figure 2). Studies have shown that in hepatitis C virus (HCV) infection, hepatic sinusoidal endothelial cells (LSECs) significantly upregulate Viperin expression through IFN-α/β autocrine signaling, which in turn inhibits viral replication [37]. STAT1 and IRF3 are key transcription factors in this pathway, which are phosphorylated and bind to the ISRE in the Viperin promoter region to drive gene expression [38]. In addition, pro-inflammatory factors such as LPS and IFN-γ synergistically enhance Viperin expression through STAT1-dependent signaling in an atherosclerosis model, suggesting its dual role in inflammatory diseases [38,39].

In addition to gene transcriptional regulation, post-translational modifications (PTMs) are another central mechanism for regulating the protein stability and antiviral activity of Viperin. Our team systematically investigated the PTM network of Viperin and revealed its dynamic regulation under viral infection and host metabolic stress. Specifically, viral or IFN stimulation catalyzes an acetylation modification of the lysine (Lys) 197 position of the Viperin protein by inducing the expression of the acetyltransferase HAT1. This modification leads to rapid degradation of Viperin protein in epithelial cells by enhancing the interaction of the ubiquitin ligase UBE4A with Viperin, prompting UBE4A to further catalyze K6-linked polyubiquitination at Lys206 of Viperin. Experiments showed that UBE4A knockdown completely restored the protein level of Viperin in epithelial cells, confirming that UBE4A is a key factor in regulating its stability [40]. Notably, the ubiquitination modification of Viperin is closely related to the dynamic balance of deubiquitination: our study demonstrated that high-salt environments weakened the host antiviral response by inhibiting the expression of the deubiquitinating enzyme USP33, leading to elevated levels of ubiquitination and reduced stability of Viperin, whereas low-salt diets significantly enhanced Viperin accumulation and antiviral function by inhibiting p97 acetylation to upregulate USP33 expression [41]. Our recent study found that when coxsackievirus B3 (CVB3) infected cardiomyocytes, the viral 3C protease blocked its ubiquitination function by cleaving UBE4A, which contributed to the abnormal accumulation of Viperin in cardiomyocytes. Excess Viperin activates the SGK1-KCNQ1 signaling axis by degrading STAT1, triggering electrophysiological disturbances in cardiomyocytes, ultimately leading to acute heart failure (AHF) [42]. This finding reveals the dual role of post-translational modifications of Viperin in viral pathogenicity—both as an antiviral effector molecule and potentially exacerbating tissue damage due to regulatory imbalance.

In addition to the classical JAK/STAT signaling pathway, the transcriptional regulation of Viperin is also regulated by other non-classical pathways, and the Toll-like receptor (TLR) signaling pathway also plays a key role in Viperin expression. For example, TLR4 activates Viperin expression through its downstream NF-κB and IRF3 pathways, a mechanism that is critical in the response to various bacterial and viral infections [43]. In addition, Viperin is also produced independently of the interferon pathway and is directly regulated by IRF1 or IRF3 [44,45]. The membrane glycoprotein of HCMV, glycoprotein B (GB), induces the phosphorylation of IRF3 and binds to the ISRE upstream of the Viperin promoter, which in turn directly initiates its transcription [46]. VSV infection stimulates IRF1 to bind to the two proximal ISREs of the Viperin promoter, which in turn activates its transcription [47]. Viperin transcription is also affected by regulatory factors such as microRNAs (miRNAs). miR-23a was found to be able to promote HSV-1 replication by downregulating the expression of IRF1, which in turn affects Viperin expression [48]. Mitochondrial antiviral signaling protein (MAVS) and peroxisomal signaling are also involved in regulation, and Viperin expression can also be directly induced by activation of MAVS and downstream IRF3 after chikungunya virus (CHIKV) infection [49]. Viperin was found to interact with PEX19 to mediate peroxisomal enhancement of the innate antiviral response [50]. In a recent study, Viperin was also found to inhibit viral translation by inducing ribosome collisions that activate the integrative stress response (ISR) pathway [34]. Together, these pathways constitute a complex regulatory network of Viperin in host defense and provide clues to further understand the molecular mechanisms of its antiviral action (Figure 2).

## 3. Mechanism of Antiviral Action of Viperin

### 3.1. Direct Antiviral Effects of Viperin

Viperin, an important interferon-stimulated gene (ISG), plays a direct antiviral role in antiviral immunity [51]. Several reports suggest that Viperin plays an important role in antiviral immunity against flaviviruses, including ZIKV, DENV, TBEV, WNV, and LGTV [13,52,53,54,55,56]. It has been shown that Viperin can interfere with the viral replication process through a variety of mechanisms and that its core function is dependent on the enzymatic activity of the S-adenosylmethionine (SAM) structural domain [57]. For example, in Zika virus (ZIKV) infection, Viperin is likely to significantly reduce viral replication efficiency by interrupting viral RNA synthesis in host cells [54,58]. Recent studies have confirmed that Viperin can interfere with early viral RNA synthesis by inducing degradation of the NS3 protein in ZIKV [53].

In addition, Viperin inhibits the assembly of viral particles by regulating the formation of host cell lipid droplets. The vMIA protein of human cytomegalovirus (HCMV) is required to bind to the N-terminal structural domain of Viperin to transport it from the endoplasmic reticulum to the mitochondria, which regulates lipid metabolism to facilitate viral assembly; if this process is blocked, the efficiency of viral replication decreases dramatically [59]. In terms of affecting lipid droplet formation and viral assembly, it was found that Viperin was able to interfere with the expression or alter the distribution of key proteins in lipid droplets, thereby disrupting the normal process of droplet formation. Viperin’s effect on lipid droplets, which are critical in viral assembly, indirectly hinders the assembly and release of viruses by providing a site of assembly and a source of energy. Viperin also targets cholesterol and interferes with its biosynthesis, which interferes with the replication of a variety of enveloped viruses [24]. In influenza virus (IAV) infection, Viperin inhibits the release of viral particles by interfering with the formation of host cell lipid rafts, a process closely related to its inhibition of farnesyl pyrophosphate synthase (FPPS) activity [14,60]. In addition, Viperin disrupts the formation of the viral replication complex by binding to the nonstructural protein NS5A of hepatitis C virus (HCV) and blocking its interaction with the host protein hVAP-33 [61,62]. It has also been shown that Viperin interferes with the viral assembly process by localizing to the endoplasmic reticulum and lipid droplet interface through its N-terminal helical structural domain [61]. These mechanisms suggest that Viperin exerts direct antiviral effects by targeting key aspects of viral replication [14,27,34] (Table 1).

### 3.2. Indirect Antiviral Effects of Viperin

The antiviral mechanism of Viperin is not limited to direct action but also includes indirect antiviral action through activation of immune signaling pathways. It has been found that Viperin activates the type I interferon (IFN-I) signaling pathway and promotes the expression of interferon-stimulated genes (ISGs). In the early stage of viral infection, Viperin activates intracellular pattern recognition receptors (PRRs) signaling pathways, such as the RIG-I-like receptor (RLR) pathway and the Toll-like receptor (TLR) pathway, after recognizing the viral pathogen-associated molecular patterns (PAMPs) [64]. Activation of these pathways prompts phosphorylation and translocation of the transcription factors IRF3 and IRF7 to the nucleus, where they bind to the relevant elements in the promoter region of the type I interferon genes, inducing the expression of type I interferons (IFN-α and IFN-β). The produced type I interferons in turn act in an autocrine and paracrine manner on the surrounding cells, activating the JAK-STAT signaling pathway and inducing the expression of more interferon-stimulated genes (ISGs), thus enhancing the body’s antiviral immune response [65]. For example, detailed analyses of the Viperin promoter sequence and transcriptional regulatory mechanisms have shown that Viperin gene expression is activated in a type I IFN signaling-dependent manner by both Toll-like receptor 3-dependent and RIG-I-independent pathways, and that the key factor regulating Viperin promoter activity is the ISGF3 complex rather than IRF3 [27,66]. In addition, Viperin amplifies the interferon response by promoting the activation of mitochondrial antiviral signaling proteins (MAVSs) and enhancing signaling in the RIG-I-like receptor (RLR) pathway [50] (Figure 2).

Viperin was shown to play a mediating role in inducing IFN-I production in plasma cell-like dendritic cells (pDCs) by mediating TLR7 and TLR9 [67]. Silencing of RASD2 reduces viability and promotes apoptosis in CD19+ B cells through inhibition of the NF-κB pathway, as well as decreasing IL-10 expression [68]. In bacterial infections, homologs of Viperin enhance the host immune response to bacterial defenses by modulating the NLR signaling pathway [69,70]. Notably, Viperin also regulates the expression of cytokines and chemokines, recruits immune cells to the site of infection, and enhances the recognition and clearance of virus-infected cells by immune cells. Viperin inhibits the phosphorylation of NF-κB and p38MAPK through activation of the AMPKα signaling pathway and reduces the release of pro-inflammatory cytokines (e.g., IL-6 and TNF-α), thereby alleviating virus-induced excessive inflammatory responses [71]. By activating the immune signaling pathway, Viperin not only enhances the antiviral capacity of host cells but also promotes the activation and recruitment of immune cells, resulting in a multi-layered immune defense.

Recent studies have also reported a novel antiviral mechanism for Viperin, whereby Viperin triggers ribosome collision-dependent translational repression to limit viral replication. The researchers found that Viperin’s free radical SAM activity is required for translational repression, which is mediated by Viperin’s enzyme product 3′-deoxy-3′,4′-didehydro-CTP (ddhCTP). Viperin triggers ribosome collisions and activates the MAPKKK ZAK pathway, which in turn activates the GCN2 arm of the integrative stress response pathway to inhibit translation. This study not only illustrates the importance of translation inhibition in the antiviral response but also identifies Viperin as a translational regulator in innate immunity [34]. These findings may provide potential new strategies to enhance the broad-spectrum antiviral capacity of the host and to improve the efficacy of IFNs in the treatment of multiple viral diseases in the clinic.

## 4. Role of Viperin in Disease

### 4.1. Viperin in Diseases Associated with Viral Infections

Viperin plays an important role in a variety of diseases associated with viral infections. In viral hepatitis, for example, it has been shown that Viperin plays a key role in the process of HCV infection [72]. The anti-HCV activity of Viperin is located at its C-terminus and interferes with its interaction with HCV NS5A by competitively interacting with the C-terminal structural domain of the host protein hVAP-33 [62]. In addition, Viperin competitively inhibits influenza virus RNA polymerase (IAV) through SAM-dependent enzymatic activity catalyzing the generation of ddhCTP, thereby limiting viral replication [15]. Studies have shown that IAV replication was significantly enhanced in MDCK cells knocked down for Viperin, while RSAD2 overexpression inhibited viral proliferation, suggesting its potential application in influenza vaccine production [73]. Viperin also inhibited NF-κB- and MAPK-mediated inflammatory responses and attenuated lung injury by activating the AMPKα signaling pathway in an influenza infection model [71]. Furthermore, in the context of human cytomegalovirus (HCMV) infection, Viperin expression may lead to enhanced infectivity, which may be achieved by altering cellular metabolism and disrupting the actin cytoskeleton [74].

The role of Viperin in COVID-19 has received much attention in recent years. Single-cell transcriptome analyses have shown abnormally high expression of Viperin in peripheral blood mononuclear cells of COVID-19 critically ill patients, which correlates with type I interferon signaling storms and may exacerbate immunopathological damage [75]. In addition, Viperin interacts with the 3′-5′ exonuclease (ExoN) of SARS-CoV-2 to block viral RNA synthesis by catalyzing ddhCTP, and mutations in ExoN attenuate this inhibitory effect [76]. Clinical cohort studies have demonstrated a positive correlation between Viperin expression levels and the rate of viral clearance in COVID-19 patients, suggesting its potential as a therapeutic target [77,78]. These studies have shown that Viperin plays a role in inhibiting viral replication and influencing the disease process in different viral infection-associated diseases through a variety of mechanisms, which is important for the occurrence, development, and prognosis of viral infectious diseases [34,77,79].

### 4.2. Potential Function of Viperin in Autoimmune Diseases

Notably, Viperin not only plays an important role in antiviral immunity but also exhibits potential functions in autoimmune diseases. Several studies have shown that the expression level of Viperin is significantly altered in autoimmune diseases such as systemic lupus erythematosus (SLE) and rheumatoid arthritis (RA) and that it exhibits bi-directional immunoregulation in autoimmune diseases, where it may influence the progression of the disease by modulating the immune response [80,81]. SLE is an autoimmune disease characterized by excessive production of IFN-I, especially IFN-α [82]. Recent studies have found that serum type I IFN levels in SLE patients are positively correlated with disease activity and that IFN-induced genes are strongly associated with the incidence of SLE [83]. In particular, the type I IFN pathway can induce autoimmunity by regulating a series of downstream genes. Therefore, it is important to study the relationship between type I IFN-induced genes and SLE [84,85,86]. Viperin has been found to be highly expressed in synovial tissues of rheumatoid arthritis (RA) patients and promotes disease progression by modulating the inflammatory response [87] and has been used clinically as a predictor of RA progression [88,89]. Furthermore, RSAD2 is significantly upregulated in atherosclerosis, and its expression is regulated by pro-inflammatory agents (i.e., lipopolysaccharides, cytomegalovirus, and IFN-γ) but not by tumor necrosis factor α or IL-1β [39].

The expression level of Viperin is also significantly increased in systemic lupus erythematosus (SLE) and correlated with the severity of the disease. RSAD2 is considered to be a key diagnostic ISG for SLE and may be useful for personalized targeted therapy for this disease [90]. A meta-analysis showed that RSAD2 had an important regulatory role in SLE patients and was significantly higher in SLE patients than in normal controls [91]. Meanwhile, some studies using gene microarrays to screen differentially expressed genes found that RSAD2 was significantly upregulated in SLE patients compared with normal controls, suggesting that the increased expression of RSAD2 may be related to the mechanism of the development of SLE and may reflect the activity of the disease to some extent [92]. Studies have shown that Viperin is highly expressed in peripheral blood mononuclear cells of SLE patients, and it reduces autoantibody production by inhibiting the TLR7/9 signaling pathway [93]. Furthermore, it has been shown that the absence of RSAD2 leads to a reduction in Th17 and Tfh cells, while the presence of RSAD2 promotes the differentiation of Th17 and Tfh cells in SLE individuals [94].

### 4.3. The Role of Viperin in Immunometabolic Reprogramming of Tumors

Metabolic reprogramming is a hallmark of malignancy that allows cancer cells to proliferate continuously using nutrients and energy [95,96,97,98]. Recent data support a role for IFN in the regulation of cancer metabolism; for example, IFN can activate the JAK/STAT signaling pathway in cancer cells to regulate metabolic processes and activate the tumor immune response [99,100,101,102]. In addition, IFN regulatory factors (IRFs) are involved in the regulation of cancer metabolism [103,104]. IFN upregulates the transcription of a large number of IFN-stimulated genes (ISGs), the products of which play a major role in the immune response [105].

It has been shown that ISG-encoded Viperin controls cancer metabolic reprogramming to promote cancer progression [106]. In recent years, it has been found that Viperin affects tumor progression by regulating lipid metabolism and glycolysis in the tumor microenvironment. This protein inhibits fatty acid β-oxidation in mitochondria, which in turn reduces ATP production and enhances glycolysis and lipogenesis during HCMV infection [46,74], suggesting that its function could be used to drive metabolic changes in cancer cells. In triple-negative breast cancer (TNBC), Viperin promotes the self-renewal capacity of tumor stem cells (CSCs) by inhibiting fatty acid β-oxidation (FAO) and enhancing lipid synthesis [106]. In addition, Viperin enhances the sugar uptake capacity of tumor cells by activating the HIF-1α signaling pathway and upregulating the expression of the glucose transporter protein GLUT1 [93]. Studies have shown that RSAD2 inhibits the proliferation and metastasis of cholangiocarcinoma (CCA) cells, suggesting that it influences tumor progression through the regulation of energy metabolism [107]. These findings reveal a complex regulatory network of Viperin in tumor immunity, and its use as a prognostic marker or therapeutic target needs to be analyzed in combination with tumor type and the immune microenvironment. The ISG-encoded protein Viperin drives metabolic alterations in support of cancer proliferation, growth, and survival and is potentially important for the development of anticancer therapies targeting the cancer metabolism and IFN response [106,108,109].

## 5. Conclusions and Perspectives

Viperin (RSAD2) is an important member of the family of interferon-stimulated genes (ISGs) that directly inhibit viral replication through multiple mechanisms. Its antiviral mechanism is mainly reflected in the direct inhibition of several aspects of viral replication, including interfering with viral RNA synthesis and affecting viral assembly and release [56]. Infection with different viruses leads to differences in RSAD2 expression levels. Some viruses induce upregulation of RSAD2 mRNA and protein expression during infection, but their antiviral activity is impaired. For example, EV71 or CSFV infection significantly increases RSAD2 mRNA and protein levels [33,110]; however, during Senecavirus A (SVA) infection, mRNA levels of RSAD2 are significantly increased, whereas its protein expression level is reduced [63]. Additionally, the HSV-1 UL41 protein significantly abrogated the antiviral activity of RSAD2 by reducing its mRNA expression level [111]. In addition, the expression level of Viperin is strongly correlated with the type I interferon signature of autoimmune diseases such as systemic lupus erythematosus (SLE) [16], and it can also be used to predict disease progression in rheumatoid arthritis (RA) [88]. Emerging evidence suggests that RSAD2 may play a role in atherosclerosis because it interferes with cellular processes that are critical in the development and progression of atherosclerosis [112]. These studies reveal that Viperin is not only a key molecule in antiviral defense but also an important bridge between innate immunity and disease pathology [113].

Based on the important role of Viperin in antiviral immunity, the development of antiviral drugs targeting the Viperin pathway is of great clinical importance. Several studies have shown that Viperin not only plays an important role in antiviral immunity but may also be a potential target for antiviral therapy [114]. Interfering peptides (IPs) are recognized as a research avenue for new drug development. Researchers found that one of these interfering peptides, VIP-IP3, effectively blocked the interaction between UBE4A and Viperin and restored the ability of epithelial cells to produce the Viperin protein, revealing a defect in the inability of epithelial cells to efficiently produce Viperin proteins and carry out their antiviral functions [40]. Recently, it was reported that the small-molecule compound L-chicoric acid (LCA) could alleviate placental vascular injury and improve pregnancy outcomes in SLE patients by inhibiting RSAD2 activity [115,116]. Moreover, RNA biomarkers based on Viperin (such as RSAD2 and IFI27) can distinguish bacterial and viral co-infections in COVID-19 patients, providing a molecular basis for precise antiviral treatment [117]. These studies indicate that Viperin may become a potential target for antiviral therapy. Through regulating the expression or activity of Viperin, it may effectively inhibit the replication and transmission of viruses, thereby achieving the therapeutic purpose.

Despite the promising potential of Viperin in antiviral and immunomodulatory applications, challenges remain in its clinical use. Firstly, the pro-inflammatory properties of Viperin may exacerbate autoimmune responses, and tissue-specific delivery systems need to be developed to balance efficacy and safety. Secondly, the metabolic regulatory function of Viperin has been shown to have a dual role in cancer; for example, high expression of RSAD2 in colorectal cancer predicts a poor prognosis, but inhibition of its activity may enhance the efficacy of immunotherapy [118]. In addition, post-translational modifications of Viperin, such as ubiquitination and acetylation, provide new targets for its dynamic regulation. For example, targeting UBE4A can stabilize Viperin protein levels to enhance antiviral responses [40].

However, there are still many unknowns in the study of Viperin, and the specific functional differences of different transcript variants as well as the precise regulatory mechanisms in different tissues and cells have yet to be thoroughly investigated. Only through a comprehensive and in-depth understanding of these aspects can we fully explore the potential of Viperin as a therapeutic target and bring breakthroughs in the diagnosis and treatment of clinical diseases. Future studies need to integrate multi-omics techniques and organoid models to elucidate the functional heterogeneity of Viperin in different microenvironments and facilitate its transformation from basic research to clinical practice. In the future, the specific action mechanism of Viperin should be further explored, and antiviral and immunomodulatory drugs targeting the Viperin pathway should be developed. Through multidisciplinary cross-innovation, Viperin is anticipated to become a “multifunctional target” for antiviral, anti-tumor, and immune disease treatments, providing novel strategies and approaches for clinical therapy.

## Figures and Tables

**Table 1:** The antiviral functions and critical domains of Viperin.

Species	Virus	Mechanism	Critical Domains	Ref.
Viruses of the genus Flavivirus	DENV	The carboxyl terminus of Viperin interacts with the NS3 protein of DENV-2 to reduce early RNA production by interfering with DENV-2 replication complex synthesis.	C′-terminus	[30,56]
	ZIKV	Interfere with early viral RNA synthesis by inducing the degradation of NS3 protein in ZIKV.	C′-terminus	[53,54]
	TBEV	Specifically degrade the nonstructural protein NS3 of TBEV and its cofactors, thereby restricting the synthesis of viral proteins of TBEV in cells and inhibiting its replication.	SAM domain	[57]
	WNV	Disruption of intracellular lipid raft synthesis.	SAM domain	[19,28]
	HCV	Interfere with the binding of NS5A protein to the host protein hVAP-33.	C′-terminus and amphipathic helix	[61,62]
	HIV	Inhibits viral egress.	SAM domain	[11]
	BUNV	Inhibition of the Bunyamwera virus replication process.	SAM domain	[26]
	EIAV	Disruption of the structure of the endoplasmic reticulum inhibits the expression of capsular membrane proteins.	N-terminal hydrophobic α-helix domain	[23]
	RABV	Disruption of the cell membrane of cholesterol and sphingolipid.	N-terminal domain	[43]
	SVA	Direct interaction between the RSAD2 aa 43–70 region and the SVA 2 C protein.	N-terminal domain	[63]
	IAV	Inhibits viral budding from the plasma membrane, possibly via interacting with FPPS.	nd	[14]

## Abbreviations

The following abbreviations are used in this manuscript:

DENV	Dengue virus
ZIKV	Zika virus
TBEV	Tick-borne encephalitis virus
WNV	West Nile virus
HCV	Hepatitis C virus
HIV	Human immunodeficiency virus
BUNV	Bunyamwera virus
EIAV	Equine infectious anemia virus
RABV	Rabies virus
SVA	Senecavirus A
IAV	Influenza A virus
